# Blood hemoglobin A1c might predict adverse differences in heart rate variability in a diabetic population: Evidence from the Midlife in the United States (MIDUS) study

**DOI:** 10.3389/fendo.2022.921287

**Published:** 2022-08-23

**Authors:** Ying Huang, Hong Chen, Dongxia Hu, Rong Wan

**Affiliations:** ^1^ Rehabilitation Department, The Second Affiliated Hospital of Nanchang University, Nanchang, China; ^2^ Cardiovascular Medicine, The Second Affiliated Hospital of Nanchang University, Nanchang, China; ^3^ Jiangxi Key Laboratory of Molecular Medicine, Nanchang, China

**Keywords:** automatic nervous system, HbA1c, heart rate variability, cardiac autonomic neuropathy, diabetes mellitus

## Abstract

**Background:**

Cardiac autonomic neuropathy in population with diabetes mellitus (DM) is frequent and linked with high risk of cardiovascular mortality. However, studies on whether blood hemoglobin A1c (HbA1c) levels are related to adverse differences in heart rate variability (HRV) in individuals with DM are scarce.

**Aim:**

We aimed to investigate the association of blood HbA1c levels with adverse differences in HRV, which is an indicator of cardiac autonomic control, in adult individuals with and without DM.

**Methods:**

Data were collected from the Midlife in the United States (MIDUS) study, and 928 individuals were analyzed for the relationship between blood HbA1c levels and HRV through a cross-sectional analysis.

**Results:**

Participants with DM had significantly higher HRV than those without DM. The smooth curve suggested inverse relationships between blood HbA1c levels and HF- and LF-HRV seen in participants with DM but not in those without DM after controlling for all covariates (age, sex, BMI, smoking, number of drinking years and exercise). Furthermore, linear regression analysis demonstrated that elevated blood HbA1c levels did contribute to adverse differences in HF-HRV (Sβ= -0.118; 95% CI -0.208, -0.027; P=0.012) and LF-HRV (Sβ= -0.097; 95% CI -0.177, -0.017; P=0.019) after controlling for these covariates in participants with DM, while in participants without DM, blood HbA1c was not significantly related to adverse differences in HF-HRV (Sβ=0.095; 95% CI -0.059, 0.248; P=0.228) or LF-HRV (Sβ=0.043; 95% CI -0.103, 0.189; P=0.565). DM has a significant modifying effect on associations between blood HbA1c and adverse differences in HF-HRV (P for interaction=0.019) and LF-HRV (P for interaction=0.029).

**Conclusions:**

We reported strong evidence that elevated blood levels of HbA1c were associated with adverse differences in HRV in the diabetic population but not in the nondiabetic population. This finding supported that long-term hyperglycemia is related to autonomic nerve injury in the diabetic population. Blood HbA1c might be a good indicator of cardiac autonomic neuropathy.

## Introduction

The increasing prevalence of diabetes mellitus (DM) in most countries contributes to an increased risk of cardiovascular (CV) events and mortality (1, 2). Diabetic autonomic neuropathy is one of the important mechanisms in the development of CV events (3–5). As an index to evaluate autonomic nervous system (ANS) function, heart rate variability (HRV) is a well-known marker for ANS dysfunction caused by an imbalance between the sympathetic and parasympathetic nervous systems (6). Previous studies have confirmed that reduced HRV has been positively associated with high CV outcomes, including incident coronary heart disease, stroke and CV mortality (7–9). HRV may represent a target for preventing CV outcomes through medication or lifestyle changes that improve or preserve ANS function (10). Importantly, the association between HRV and CV outcomes has been shown to be stronger in the DM population (8, 9). A recent study also showed that reduced HRV may exist in the course of prediabetes (11). However, the clinical value of risk factors for identifying individuals at high risk of ANS dysfunction is unknown.

Glycosylated hemoglobin is the product of the combination of hemoglobin in red blood cells and sugars in blood through nonenzymatic reactions (12). The concentration of glycosylated hemoglobin can effectively reflect the average blood glucose level in the previous 8-12 weeks (13). Glycosylated hemoglobin consists of HbA1a, HbA1b and HbA1c. HbA1c accounts for approximately 70% of all glycosylated hemoglobin and is commonly used as a monitoring index for diabetes control (14, 15). To date, few studies have investigated the association between blood HbA1c levels and HRV. We analyzed data from the Midlife in the United States (MIDUS) study to estimate the association between blood HbA1c levels and HRV in a general adult population. The MIDUS study provided enough information on cardiac function evaluation and blood tests. Therefore, we fully investigated the relationship between blood concentrations of HbA1c and HRV in the adult population.

## Methods

### Study participants

This is a retrospective analysis on data from the MIDUS study conducted with 7,108 noninstitutionalized adults in 1995 (16). The MIDUS II study with 4,963 participants was the first follow-up between 2004 and 2006 (17). A total of 1255 participants in MIDUS II took part in a biomarker study (18). Each participant was invited to attend a clinical research center (the University of Wisconsin, University of California, Los Angeles or Georgetown University), and trained medical staff performed a comprehensive examination including blood biochemical markers, a comprehensive physical exam and medical history data collection (18). Of the study participants with blood biomarker data (N=1,225), some subjects had missing data (N=327). As such, our study finally included 928 adults by using a cross-section analysis. Consistent with the Declaration of Helsinki, IRB approval was obtained for data collection for the MIDUS 2 Biomarker Project at the three sites, and written consent was collected from each participant. The data used in the present study are publicly available through the Inter-University Consortium for Political and Social Research (ICPSR). All analyses were conducted in accordance with the preregistration.

### Assessment of HRV

After an overnight stay at the clinical research centers, all participants were given a light breakfast without caffeine. Then, the HRV psychophysiology protocol was performed for these participants after breakfast. Electrocardiogram (ECG) electrodes were placed on the two shoulders (right and left) and the left lower quadrant. Subsequently, HRV data from this resting baseline were recorded. Analog ECG signals were digitized at 500 Hz by a 16-bit A/D conversion board (National Instruments, Austin, TX) and passed to a microcomputer. The ECG waveform, submitted to an R-wave detection routine, was performed by custom-written software, producing an RR interval series. Errors caused by marking R-waves were revised by visual inspection. Interpolation was used for correcting ectopic beats. HF-HRV and LF-HRV were computed using an interval method for computing Fourier transforms (19). The mean value of the two measurements was calculated and further analyzed. A higher HRV represented better ANS function.

### Blood biomarkers

In summary, blood samples were obtained from three examination sites (the University of Wisconsin, University of California, Los Angeles and Georgetown University). All participants underwent fasting blood sampling before breakfast, and these blood samples were analyzed in the MIDUS Biocore Lab. The glycated hemoglobin assays were performed at Meriter Labs (Madison, WI) using a Cobas Integra analyzer (Roche Diagnostics, Indianapolis, IN). See Ryff and colleagues for more details about the assessment of these blood biomarkers.

### Confounding variables

The following variables were included as covariates (each measured at the biomarker clinic visit): age, sex (male, female), body mass index (BMI), smoking status, number of drinking years, and chronic conditions [history of diabetes, hypertension, heart disease and transient cerebral ischemia (TIA) or stroke]. Smoking status was dichotomized as “ever smoker” or “nonsmoker”. Exercise was defined as “whether or not the frequency of exercise was ≥ 3/week”. Chronic conditions for the participants who had ever had CVDs were classified as “yes” or “no”. BMI was measured as weight (kg) divided by the square of height (cm).

### Statistical analysis

Statistical analyses in the current study were performed by using EmpowerStats 3.0. A P value less than 0.05 was defined as a significant difference. All analyses were first conducted on participants with and without DM. The Mann -Whitney U test and chi-square test were used for comparisons between the two groups. Then, logarithmic transformation was conducted on both HF-HRV and LF-HRV values to reduce skewed distributions. Single-factor smoothing analysis was performed to assess the association between blood HbA1c levels and HRV. A multivariate linear regression model was further established to estimate the independent effect of blood HbA1c on adverse differences in HRV. Each model reported a standardized correlation coefficient (Sβ), and confidence intervals (CIs) had thresholds of 95%.

In the calibration analysis, blood HbA1c was assessed as an independent predictor for adverse differences in HRV. The details are as follows: 1) The crude model tested the effects of nonadjustment on adverse differences in HRV. Model 1 included age and sex. Model 2 included Model 1 and health-related confounders (BMI, smoking, number of drinking years and exercise). Similarly, a corrected smooth curve analysis was established to analyze the independent effect of blood HbA1c on adverse differences in HRV. Specifically, subgroup analyses further examined the effects of chronic conditions (history of hypertension, heart disease, and TIA or stroke) on the association of blood HbA1c with HRV. Similar to the Model 2 analysis, each subgroup analysis adjusted for age, sex, BMI, smoking, number of drinking years and exercise.

## Results

### Characteristics of participants

A total of 928 adult participants were analyzed for HF-HRV and LF-HRV as well as blood HbA1c. A total of 11.6% of these study participants were diagnosed with DM. Demographic data on participants with DM (N=128) and without DM (N=820) are shown in **Table 1**. Both groups did not significantly differ in sex, smoking status, number of drinking years, HF-HRV and history of TIA or stroke. DM participants had significantly higher age and BMI; higher rates of heart disease, hypertension and cholesterol problems; and a lower frequency of exercise and HF-HRV compared with those without DM.

**Table 1:** Characteristics of participants (N=928).

Variables	With DM (N=108)	Without DM (N=820)	P value
Age (years)	55 (48-62)	53 (44-61)	0.015
Gender (male), n (%)	49 (45.37)	373 (45.49)	0.982
BMI (kg/m2)	31.74 (28.39-35.62)	28.03 (25.01-32.32)	<0.001
Ever smoker, n (%)	59 (54.63)	415 (50.61)	0.432
Number of drinking years	5 (2-18.25)	5 (2-14.62)	0.127
Frequency of exercises ≥3/ week, n (%)	74 (68.52)	636 (77.56)	0.037
Natural log of LF-HRV (0.04-0.15 Hz)	5.32 (4.54-5.93)	5.81 (4.91-6.56)	<0.001
Natural log of HF-HRV (0.15-0.50 Hz)	4.88 (4.00-5.65)	4.99 (4.05-5.75)	0.237
**CVD history**			
Heart disease, n (%)	18 (16.67)	66 (8.05)	0.003
Hypertension, n (%)	64 (59.26)	254 (30.98)	<0.001
TIA or stroke, n (%)	6 (5.56)	24 (2.93)	0.147
Cholesterol problems, n (%)	68 (62.96)	326 (39.76)	<0.001
**Blood analysis**			
HemoglobinA1c (%)	7.13 (6.37-8.78)	5.74 (5.50-6.04)	<0.001
Fasting Glucose (mg/dL)	117.00 (104.00-153.00)	95 (89.00-102.00)	<0.001
Fasting Insulin (uIU/mL)	15.50 (10.00-24.25)	9.00 (6.00-16.00)	<0.001

M (IQR) for non-normally distributed variables, and n (%) for categoric variables.

DM, diabetes mellitus; BMI, body mass index; LF-HRV, Low frequency-heart rate variability; HF-HRV, High frequency heart rate variability; CVD, cardiovascular disease; TIA, transient ischemic attack.

### Elevated blood HbA1c levels contributed to adverse differences in HRV

**Figure 1:**
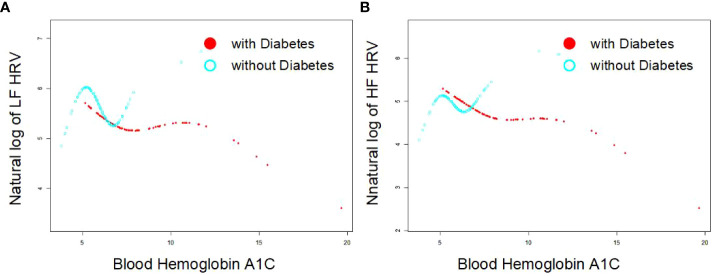
Single exponential smooth curve for the association between blood HbA1c and HRV [As **(A, B)** indicates, the single-factor smooth curve shows inverse relationships between blood HbA1c levels and HF-HRV and LF-HRV values in participants with DM (P<0.05) but not in those without DM (P>0.05)].

**Figure 2:**
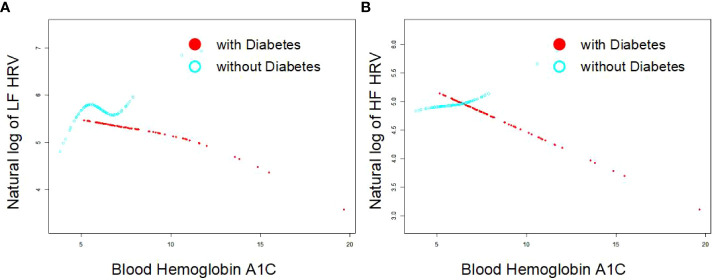
Corrected smooth curve for the association between blood HbA1c and HRV [As **(A, B)** indicates, the corrected smooth curve suggested inverse relationships between blood HbA1c levels and HF-HRV and LF-HRV values in participants with DM (P<0.05) but not in those without DM (P>0.05)after controlling for all covariates, including age, sex, BMI, smoking, number of drinking years and exercise].

As **Figures 1A, B** indicates, the single-factor smooth curve shows inverse relationships between blood HbA1c levels and HF-HRV and LF-HRV values in participants with DM but not in those without DM. After controlling for all covariates, including age, sex, BMI, smoking, number of drinking years and exercise, the corrected smooth curve suggested similar results that significant relationships between blood HbA1c levels were seen in participants with DM but not in those without DM (**Figures 2A, B**).

Furthermore, linear regression analysis demonstrated that elevated blood HbA1c levels did contribute to adverse differences in HF-HRV (Sβ=-0.118; 95% CI -0.208, -0.027; P=0.012) and LF-HRV (Sβ=-0.097; 95% CI -0.177, -0.017; P=0.019) in Model 2 after controlling for all covariates in participants with DM, as shown in **Table 2**. In participants without DM, blood HbA1c was not significantly related to adverse differences in HF-HRV (Sβ=0.095; 95% CI -0.059, 0.248; P=0.228) or LF-HRV (Sβ=0.043; 95% CI -0.103, 0.189; P=0.565).

**Table 2:** Multiple linear regression analysis for relationship between blood hemoglobinA1c and HRV.

	LF-HRV			HF-HRV		
Variables	Sβ	95% CI	*P* Value	Sβ	95% CI	*P* Value
**With DM**						
Crude Model	-0.075	(-0.162, 0.013)	0.099	-0.114	(-0.206, -0.021)	0.017
Model 1	-0.094	(-0.174, -0.014)	0.023	-0.120	(-0.213, -0.028)	0.012
Model 2	-0.097	(-0.177, -0.017)	0.019	-0.118	(-0.208, -0.027)	0.012
**Without DM**						
Crude Model	-0.238	(-0.387, -0.090)	0.002	-0.046	(-0.196, 0.103)	0.543
Model 1	-0.024	(-0.167, 0.118)	0.737	0.103	(-0.046, 0.252)	0.176
Model 2	0.043	(-0.103, 0.189)	0.565	0.095	(-0.059, 0.248)	0.228

**Crude Model :** No adjustment.

**Model 1:** Adjusted for age and gender.

**Model 2:** Adjusted for age, gender, BMI, ever smoker, number of drinking years and exercise.

DM, diabetes mellitus; LF-HRV, low frequency-heart rate variability; HF-HRV, High frequency heart rate variability; BMI, body mass index.

### Subgroup analyses for the association of blood HbA1c with HRV

Subgroup analyses were further evaluated for the effects of chronic conditions (diabetes, hypertension, heart disease and TIA or stroke) on the association of blood HbA1c with HRV. As shown in **Table 3**, we found that DM had a significant modifying effect on associations between blood HbA1c and adverse differences in HF-HRV (P for interaction=0.019) and LF-HRV (P for interaction=0.029). The results further clarified the differences in the association between blood HbA1c and HRV among diabetic participants. However, we did not observe that hypertension, heart disease and TIA or stroke had no modifying effect on the association.

**Table 3:** Subgroup analyses for association between blood hemoglobinA1c and HRV by adding “CVD history” as stratification variables.

Variables	n	LF-HRV				HF-HRV				
		Sβ	95% CI	*P* Value	*P #*		Sβ	95% CI	*P* Value	*P #*
Ever had DM (YES)	108	-0.097	(-0.177, -0.017)	0.019	0.029		-0.118	(-0.208, -0.027)	0.012	0.019
Ever had DM (NO)	820	0.043	(-0.103, 0.189)	0.565			0.095	(-0.059, 0.248)	0.228	
									
Ever had heart disease (YES)	84	-0.102	-0.337, 0.134	0.392	0.901		-0.058	-0.305, 0.189	0.643	0.916
Ever had heart disease (NO)	884	-0.091	-0.154, -0.029	0.004			-0.072	-0.138, -0.006	0.032
									
Ever had hypertension (YES)	318	-0.133	-0.239, -0.027	0.014	0.419		-0.102	-0.210, 0.007	0.066	0.854
Ever had hypertension (NO)	610	-0.047	-0.122, 0.027	0.210			-0.043	-0.122, 0.035	0.278
									
Ever had TIA or stroke (YES)	30	-0.468	-0.882, -0.053	0.029	0.063		-0.343	-0.743, 0.058	0.090	0.172
Ever had TIA or stroke (NO)	898	-0.070	-0.132, -0.009	0.025			-0.044	-0.109, 0.021	0.185
									
Ever had cholesterol problems (YES)	394	-0.166	-0.210, -0.023	0.015	0.904		-0.095	-0.194, 0.004	0.059	0.464
Ever had cholesterol problems (NO)	534	-0.059	-0.140, 0.022	0.156			-0.023	-0.109, 0.062	0.593

Adjusted for age, gender, BMI, ever smoker, number of drinking years and exercise.

P#: P interaction.

DM, diabetes mellitus; CVD, cardiovascular disease; LF-HRV, low frequency-heart rate variability; HF-HRV, High frequency heart rate variability; TIA, transient ischemic attack; BMI, body mass index.

## Discussion

In this cross-sectional study in an adult population, our findings were that diabetic patients demonstrated a significant decrease in LF-HRV and HF-HRV. We observed that elevated blood levels of HbA1c are strongly and negatively related to adverse differences in HF-HRV and LF-HRV in individuals with DM but not in individuals without DM, which may be explained by the deleterious metabolic effects of hyperglycemia on cardiac autonomic nerves. The associations remained significant after adjustment for demographic characteristics and lifestyle (age, sex, BMI, smoking, number of drinking years and exercise) in the diabetic population. Controlling blood glucose levels so that they are stable and at normal levels might improve ANS dysfunction in the diabetic population, which is beneficial to prevent adverse CV events.

ANS dysfunction is characterized by decreased parasympathetic activity and increased sympathetic activity, indicating a need for vigilance of chronic diseases (20). Importantly, common chronic diseases, including cancer, rheumatoid arthritis, myocardial infarctions, obesity and metabolic syndrome, hypertension and depression, are related to elevated sympathetic activity and reduced parasympathetic activity (21–26). Previous studies have also shown that diabetes has an adverse effect on HRV (5, 27). One study reported that elderly individuals with DM may have worse parasympathetic control (27). Another meta-analysis involving 25 case-control studies with 2,932 type 2 diabetes patients suggested that blood glucose and HbA1c were associated with several HRV parameters (5). Consistently, our results again demonstrated that high blood HbA1c levels contributed to an increased risk of adverse differences in HRV in subjects with DM. In contrast, we observed that the association was not significant in individuals without DM. A reasonable explanation may be that DM is a metabolic disease responsible for cardiac autonomic neuropathy that affects both sympathetic and parasympathetic fibers, reflecting the fact that DM leads to ANS dysfunction.

Interestingly, our results showed that blood glucose levels were not associated with LF-HRV and HF-HRV in DM or non-DM participants, which seems contradictory to the above results (**Supplementary Materials Table 1**). A possible explanation for this uncorrelated association is that blood glucose levels are not truly a stable biomarker to evaluate the degree of diabetes control compared with blood HbA1c. A single blood glucose test only reflects the immediate blood glucose situation, which is affected by many factors, such as recent diet, exercise and medicines. HbA1c reflects the level of blood glucose in the previous 2-3 months, and the interference factors are relatively small, which can better reflect the overall control of diabetes (13–15). In fact, our study participants were from different American communities. Although their physical condition was good, participants with DM or chronic diseases were treated with medicines, which may be a powerful explanation for our contradictory results. The association of blood HbA1c with LF- and HF-HRV is logical in DM participants.

More interestingly, we further observed that blood insulin levels were related to reduced LF-HRV and HF-HRV in DM and non-DM participants, respectively (**Supplementary Materials Table 2**). Blood insulin, the main hormone that reduces blood glucose in the body, is an important indicator for the diagnosis and typing of diabetes. Its level in the blood is closely related to the degree of blood glucose control. A clinical study showed that parasympathetic activity can be triggered by elevated blood levels of glucose *via* increased insulin secretion in a healthy population (28). This may partly explain our results of why blood insulin levels are closely related to HRV in non-DM participants.

To the best of our knowledge, few data have been reported on the association between dyslipidemia and HRV in the literature. Several studies have shown that lowering blood low-density lipoprotein (LDL) levels by statin therapy contributes to beneficial effects in HRV parameters (29, 30). In our study, although a history of cholesterol problems had no interaction effect on blood HbA1c with LF- and HF-HRV, elevated blood HbA1c levels were significantly related to adverse differences in HRV in participants with a history of cholesterol problems rather than those who had never had cholesterol problems, which was consistent with two previous results (29, 30). Previous studies reported that increased blood pressure was associated with decreased HRV (5, 31) and that impaired ANS function precedes the progression of clinical hypertension (32). Coincidentally, high blood HbA1c levels were more significantly linked with adverse differences in HRV in participants with a history of hypertension than in participants without a history of hypertension. However, we did not observe a significant association between blood HbA1c and HRV in participants with a history of heart disease, which has rarely been reported in the literature. Autonomic nervous system dysfunction caused by organic lesions of the heart may explain this phenomenon. Moreover, we found that a history of TIA or stroke had no impact on the relationship, which may be related to the fact that autonomic nervous system function is not closely related to cerebrovascular diseases.

There are several strengths in our study. This study had a large sample size from the general population and a community-based design, which provided high-quality blood HbA1c and HRV data. Blood HbA1c can reflect diabetes control over the previous few months. Repeating HRV measurements allowed us to adjust HRV error. Several previous studies have shown that BMI, exercise and other living habits were associated with HRV (33, 34). We adjusted for demographic characteristics (age and sex) and lifestyle (BMI, smoking, drinking and exercise), providing a more reliable association between blood HbA1c and HRV. Moreover, subgroup analysis by using “CVDs” as covariates determined whether CVDs had an obvious modifying effect on the independent relationship.

There were several notable limitations in the present study. First, due to the inherent disadvantages of cross-sectional analysis in establishing causality, it was difficult to confirm whether elevated blood HbA1c precedes adverse differences in HRV or impaired ANS function. Second, all participants who took part in this study needed to be relatively healthy to go to the three clinical research centers of the MIDUS study. Some individuals with a history of serious diseases were not included; thus, this may have led to potential selection bias. Third, all recruited participants with a history of DM were included in this study, but the specific type of diabetes, duration of the disease and medications were unclear. We could not rule out the interference of these confounding factors on the relationship between blood HbA1c and HRV. Fourth, the variables (history of CVDs) used for subgroup analysis mainly came from participants’ self-reports by a questionnaire survey; thus, undiagnosed disease history in many participants might cause some error in the results. Fifth, of all participants with blood biomarker data (N=1,225), only 928 study samples were analyzed in our study, and 327 individuals who had missing data were not further analyzed. Sixth, there is established evidence that LF-HRV is poor indicator of sympathetic nervous system (SNS) activity (35, 36), which are inconsisitent with our results. More studies are needed to resolve this controversy in the future. Finally, the lack of an association between HRV and HbA1C in the non-DM group could easily be due to the constricted range of HbA1c in this group. By definition, non-DM people will tend to have much lower HbA1C values, which is evident in the shorter line in **Figures 1** and **2**. Therefore, diabetes-related differences on the association between HRV and HbA1C need to be further clarified.

We first reported that elevated blood HbA1c levels contributed to adverse differences of HRV in the diabetic population but not in the nondiabetic population. The detrimental effects of impaired glucose metabolism on HRV suggested that blood HbA1c might be a potential biomarker for predicting cardiac autonomic neuropathy.

## Data availability statement

The original contributions presented in the study are included in the article/**Supplementary Material**. Further inquiries can be directed to the corresponding authors.

## Ethics statement

The studies involving human participants were reviewed and approved by Consistent with the Declaration of Helsinki, IRB approval was obtained for data collection for the MIDUS 2 Biomarker Project at the three sites, and written consent was collected from each participant. The patients/participants provided their written informed consent to participate in this study.

## Author Contributions

YH and HC completed data analysis and first draft writing. DH and RW completed data supervision and modification. All authors contributed to the article and approved the submitted version.

## Funding

This work was supported by grants from the National Natural Science Foundation of China (NSFC 82060059) and the General Project of the Jiangxi Natural Science Foundation (20212BAB206055).

## Conflict of interest

The authors declare that the research was conducted in the absence of any commercial or financial relationships that could be construed as a potential conflict of interest.

## Publisher’s note

All claims expressed in this article are solely those of the authors and do not necessarily represent those of their affiliated organizations, or those of the publisher, the editors and the reviewers. Any product that may be evaluated in this article, or claim that may be made by its manufacturer, is not guaranteed or endorsed by the publisher.
