# Supplementary material for: Blood hemoglobin A1c might predict adverse differences in heart rate variability in a diabetic population: Evidence from the Midlife in the United States (MIDUS) study
Source: Front Endocrinol (Lausanne). 2022 Aug 23;13:921287. doi: 10.3389/fendo.2022.921287 (PMC9446475; doi:10.3389/fendo.2022.921287)
Supplement: Supplementary file 1 [file DataSheet_1.docx]

| **Supplementary materials Table 1.** Multiple linear regression analysis for relationship between blood fasting glucose levels and HRV | | | | | | |
| --- | --- | --- | --- | --- | --- | --- |
|  | **LF-HRV** | | | **HF-HRV** | | |
| Variables | Sβ | 95% CI | *P* Value | Sβ | 95% CI | *P* Value |
| **With DM** |  |  |  |  |  |  |
| Crude Model | -0.008 | (-0.090, 0.075) | 0.857 | -0.014 | (-0.102, 0.073) | 0.748 |
| Model 1 | -0.037 | (-0.114, 0.039) | 0.338 | -0.019 | (-0.108, 0.071) | 0.682 |
| Model 2 | -0.039 | (-0.116, 0.038) | 0.325 | -0.009 | (-0.097, 0.079) | 0.844 |
| **Without DM** |  |  |  |  |  |  |
| Crude Model | -0.114 | (-0.265, 0.037) | 0.140 | -0.095 | (-0.247, 0.056) | 0.218 |
| Model 1 | -0.096 | (-0.237, 0.046) | 0.187 | -0.039 | (-0.187, 0.110) | 0.609 |
| Model 2 | -0.033 | (-0.180, 0.113) | 0.654 | -0.055 | (-0.209, 0.098) | 0.480 |
| **Crude Model :** No adjustment.  **Model 1:** Adjusted for age and gender.  **Model 2:** Adjusted for age, gender, BMI, ever smoker, number of drinking years and exercise.  DM, diabetes mellitus; LF-HRV, low frequency-heart rate variability; HF-HRV, High frequency heart rate variability; BMI, body mass index | | | | | | |

| **Supplementary materials Table 2.** Multiple linear regression analysis for relationship between blood fasting insulin levels and HRV | | | | | | |
| --- | --- | --- | --- | --- | --- | --- |
|  | **LF-HRV** | | | **HF-HRV** | | |
| Variables | Sβ | 95% CI | *P* Value | Sβ | 95% CI | *P* Value |
| **With DM** |  |  |  |  |  |  |
| Crude Model | -0.153 | (-0.249, -0.057) | 0.002 | -0.192 | (-0.292, -0.091) | <0.001 |
| Model 1 | -0.166 | (-0.253, -0.079) | <0.001 | -0.203 | (-0.304, -0.102) | <0.001 |
| Model 2 | -0.185 | (-0.272, -0.097) | <0.001 | -0.218 | (-0.317, -0.119) | <0.001 |
| **Without DM** |  |  |  |  |  |  |
| Crude Model | -0.144 | (-0.227, -0.060) | <0.001 | -0.111 | (-0.195, -0.027) | 0.001 |
| Model 1 | -0.185 | (-0.262, -0.109) | <0.001 | -0.134 | (-0.215, -0.053) | 0.001 |
| Model 2 | -0.153 | (-0.238, -0.068) | <0.001 | -0.170 | (-0.260, -0.081) | <0.001 |
| **Crude Model :** No adjustment.  **Model 1:** Adjusted for age and gender.  **Model 2:** Adjusted for age, gender, BMI, ever smoker, number of drinking years and exercise.  DM, diabetes mellitus; LF-HRV, low frequency-heart rate variability; HF-HRV, High frequency heart rate variability; BMI, body mass index | | | | | | |
